# UNC-108/RAB-2 is required for *C. elegans* stress-induced sleep

**DOI:** 10.17912/micropub.biology.000112

**Published:** 2019-04-26

**Authors:** Bryan Robinson, Cheryl Van Buskirk

**Affiliations:** 1 Department of Biology, California State University Northridge, Northridge, CA 91330

**Figure 1:**
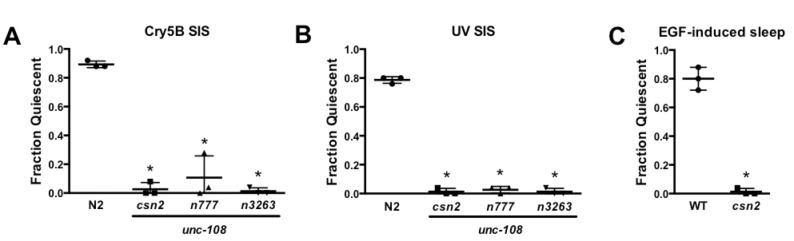
UNC-108 is required for stress-induced sleep (SIS) and acts downstream of EGF signaling. **(A,B)** Compared to wild-type N2, *unc-108(lf)* mutants are defective for SIS induced by Cry5B toxin or UV light (P< 0.0001, one-way ANOVA with Dunnett’s multiple comparisons test). Animals were exposed to Cry5B-expressing bacteria as described (Hill et al., 2014) and examined 20 min later, or exposed to UV radiation as described (Goetting et al., 2017) and examined 60 min later. These time points have been shown to be associated with robust ALA-dependent quiescence (Hill et al., 2014; Goetting et al., 2018). **(C)** LIN-3/EGF overexpression induces sleep in wild type but not in *unc-108(csn2)* animals (P< 0.0001, two-tailed Fisher’s exact test). EGF overexpression from a *hs:lin-3* transgene (Van Buskirk and Sternberg, 2007) was induced by mild heat shock (33°C for 10 min) and animals were examined 60 min later for quiescence. In all panels, quiescence was defined as a complete cessation of locomotion, head movement and pharyngeal pumping during a 5 sec examination on a stereomicroscope. Mean and SD of three independent trials are shown, and each data point represents one trial of 25 well-fed young adult animals.

## Description

In a genetic screen for mutants defective in stress-induced sleep (SIS) we isolated *csn2*, an allele of the UNC-108/RAB-2 GTPase. The point mutation in *unc-108(csn2)* is identical to that in the previously characterized loss-of-function allele *unc-108(n3263),* substituting a glutamine in place of a glycine that is conserved among Ras superfamily members (Mangahas et al., 2008). Similar to other *unc-108(lf)* mutants, *csn2* animals move slowly (not shown). Here we show that *unc-108(csn2)* as well as previously characterized *unc-108* alleles are SIS-defective (Panels A, B). While the majority of wild-type N2 animals cease head movement, locomotion and pharyngeal pumping following exposure to damaging conditions, *unc-108(lf)* animals retain all of these activities. This coordinated impairment of sleep-associated behaviors argues against a role for UNC-108 downstream of the SIS-promoting ALA neuron, which acts via the collective action of neuropeptides with overlapping but distinct effects on the sub-behaviors of sleep (Nath et al., 2016).

SIS is dependent on Epidermal Growth Factor Receptor (EGFR) activation within ALA, and sleep can be triggered not only by noxious conditions but also by forced overexpression of LIN-3/EGF (Van Buskirk and Sternberg, 2007). We found that *unc-108(csn2)* animals are resistant to EGF-induced sleep (Panel C), indicating that UNC-108 functions downstream of EGF signaling within the SIS pathway. Together these results suggest that UNC-108 functions within ALA.

UNC-108 is widely expressed within the *C. elegans* nervous system and is implicated in the recycling of receptors through the endocytic pathway (Chun et al., 2008) as well as in dense core vesicle (DCV) maturation (Sumakovic et al., 2009; Edwards et al., 2009). We speculate that the UNC-108 SIS defect arises from deficits in EGFR trafficking or in the maturation of DCVs within the ALA neuron.

## Reagents

Strains available from the CGC: N2 Bristol, MT1656*unc-108(n777)*, ZH382 *unc-108(n3263)*, PS5970 *syIs197[hs::LIN-3c(cDNA) + myo-2p::DsRed + pha-1(+)];him-5*. Strains available upon request: CVB30 *unc-108(csn2)*, CVB31*syIs197;unc-108(csn2)*.
